# Phylogenomic analyses across land plants reveals motifs and coexpression patterns useful for functional prediction in the BAHD acyltransferase family

**DOI:** 10.3389/fpls.2023.1067613

**Published:** 2023-02-10

**Authors:** Lars H. Kruse, Benjamin Fehr, Jason D. Chobirko, Gaurav D. Moghe

**Affiliations:** ^1^ Plant Biology Section, School of Integrative Plant Science, Cornell University, Ithaca, NY, United States; ^2^ Michael Smith Laboratories, University of British Columbia, Vancouver, BC, Canada; ^3^ Computational Biology Department, Cornell University, Ithaca, NY, United States; ^4^ Molecular Biology and Genetics Department, Cornell University, Ithaca, NY, United States

**Keywords:** gene family, phylogenomics, protein function, co-expression, plant metabolism

## Abstract

The BAHD acyltransferase family is one of the largest enzyme families in flowering plants, containing dozens to hundreds of genes in individual genomes. Highly prevalent in angiosperm genomes, members of this family contribute to several pathways in primary and specialized metabolism. In this study, we performed a phylogenomic analysis of the family using 52 genomes across the plant kingdom to gain deeper insights into its functional evolution and enable function prediction. We found that BAHD expansion in land plants was associated with significant changes in various gene features. Using pre-defined BAHD clades, we identified clade expansions in different plant groups. In some groups, these expansions coincided with the prominence of metabolite classes such as anthocyanins (flowering plants) and hydroxycinnamic acid amides (monocots). Clade-wise motif-enrichment analysis revealed that some clades have novel motifs fixed on either the acceptor or the donor side, potentially reflecting historical routes of functional evolution. Co-expression analysis in rice and Arabidopsis further identified BAHDs with similar expression patterns, however, most co-expressed BAHDs belonged to different clades. Comparing BAHD paralogs, we found that gene expression diverges rapidly after duplication, suggesting that sub/neo-functionalization of duplicate genes occurs quickly *via* expression diversification. Analyzing co-expression patterns in Arabidopsis in conjunction with orthology-based substrate class predictions and metabolic pathway models led to the recovery of metabolic processes of most of the already-characterized BAHDs as well as definition of novel functional predictions for some uncharacterized BAHDs. Overall, this study provides new insights into the evolution of BAHD acyltransferases and sets up a foundation for their functional characterization.

## 1 Introduction

The metabolic diversity of plants is immense, and this diversification is a result of frequent gene duplications in plant genomes as well as enzyme promiscuity (Moghe and Kruse, 2018; Pichersky and Raguso, 2018). Proliferation of duplicated genes *via* tandem, segmental and whole genome duplication has resulted in the emergence of enzyme families, which abound in plant metabolism. Reduced selection pressure, reduced turnover rates, and increased promiscuity are key characteristics of such families (Milo and Last, 2012). Gene duplication and divergence has also driven the creation in emergence of novel clades especially in larger families. Understanding how these clades originated and evolved is crucial to understanding how new functions emerge in enzyme families. In this study, we sought to examine the patterns of sequence and expression evolution in the different clades of the BAHD acyltransferase enzyme family (BAHDs or BAHD family) across plant evolution.

The BAHD family is one of the largest multi-functional enzyme families in plants (D’Auria, 2006; Bontpart et al., 2015; Kruse et al., 2022; Moghe et al., 2023). Members of the family perform an acylation reaction using an acyl coenzyme A donor and an acceptor with a hydroxyl or an amine group. The acyl chains transferred can be very diverse and can include aromatic groups (e.g. benzoyl, coumaroyl) as well as aliphatic chains from 2-12 carbons long, with unsaturation (e.g. tigloyl) and branching (e.g. isovaleryl). The family comprises members involved in a wide range of metabolic pathways such as those of lignins, cuticular waxes, anthocyanins and flavonoids, herbivore defense compounds, polyamines, volatile terpenes, aromatics, and many others (D’Auria, 2006; Bontpart et al., 2015; Kruse et al., 2022; Moghe et al., 2023). Thus, the BAHD family has played a critical role in adaptation of plants to terrestrial environments, abiotic stresses and biotic interactions. The wide range of decorations performed by many substrate-promiscuous members of this family leads to emergence of new structural scaffolds (e.g. monolignols, acylsugars) or altered physicochemical properties (e.g. aromatic esters, acylated anthocyanins), increasing the functional diversity of plant metabolites.

BAHDs are closely related to alcohol acyltransferases in fungal species (Moghe et al., 2023) and our previous study (Kruse et al., 2022) revealed that this family expanded in land plants from 1-2 members in algae to ~100 members in several diploid angiosperm genomes, likely *via* tandem gene duplication. Eight clades were identified in the family of which seven (clades 1-7) are present across land plants and clade 0 present only in algae. While most clades comprise enzymes restricted to using a predictable substrate class (e.g. aromatic or aliphatic alcohols, anthocyanins/flavonoids), some clades have diversified members and more lineage-specific sub-clades. Prior studies have also identified rapid functional divergence in BAHDs, even between species (Fan et al., 2016; Fan et al., 2017) and populations (Kim et al., 2012; Schilmiller et al., 2015; Landis et al., 2021) thereby revealing a substantial diversification of the BAHD family in land plants. A recent review described the mechanistic and evolutionary aspects of BAHDs in detail (Moghe et al., 2023). However, the clade-wise patterns of evolution in this family in land plants have not been studied, limiting our understanding of rapid enzyme diversification in such a large and important enzyme family.

In this study, we sought to determine the different ways by which BAHDs have diversified at the sequence, structural, and expression level during land plant evolution. We found evidence of clade-specific expansions and fixation of lineage-specific clades at different points in the evolution of plants. Discriminant analysis of clade-specific motifs revealed some clades with acceptor-side evolution vs. others with donor-side evolution. We also found that duplicated BAHDs have diversified at both expression and substrate-preference level, although some still retain functional similarity with their closest paralogs. Overall, this study provides novel insights into the emergence of functional diversity in the BAHD acyltransferase family.

## 2 Materials and methods

### 2.1 Identification of BAHD proteins from sequenced proteomes and analysis of genomic features

For the identification of BAHD acyltransferases from the genomes analyzed in this study (**Supplementary File 3**) we followed the same approach used previously (Kruse et al., 2022), specifically, using the PFAM domain PF02458 with the HMMER software (Potter et al., 2018). After identification, we gathered additional genomic information from the respective general feature format (GFF) files for each species using custom Python scripts.

### 2.2 Motif analysis

We identified the top five enriched motifs in each clade using discriminant analysis *via* STREME v5.4.1 (Bailey, 2021) using default parameters but with following modifications: *–protein -nmotifs 5*. We used sequences from each clade as well as orthologous sequences (OGs) corresponding to those clades, which had been previously defined (Kruse et al., 2022) using OrthoFinder (Emms and Kelly, 2019). As background distribution, we used all other BAHD sequences not assigned to that specific clade. Protein structures were downloaded from the Protein Data Bank (Berman et al., 2000) or the AlphaFold protein structure database (for AtCER2 only) (https://alphafold.ebi.ac.uk/). The top 5 motifs were mapped onto the structures using the UCSF Chimera software (Pettersen et al., 2004). Whether the motifs were exposed to the acceptor/donor binding domains was determined manually based on knowledge of these regions as per the AtHCT and Dm3MAT3 structures.

### 2.3 Phylogenetic analysis

To generate species-specific phylogenetic trees, a protein sequence alignment of all identified BAHDs was generated using MAFFT v.7.453-with-extensions as described earlier (Kruse et al., 2022). IQ-Tree v1.6.10 (Nguyen et al., 2015) was then used to infer a phylogenetic tree using following parameters: *-st AA -nt AUTO -ntmax 12 -b 1000 -m TEST* with automatic model selection. The resulting trees were visualized using iTol v.5.6.2 (Letunic and Bork, 2021).

### 2.4 Blast and phmmer sequence mapping

To map the known BAHD clades (Kruse et al., 2022) to the BAHD sequences identified from the different analyzed species, we used two different approaches. In approach 1 we used blastp to map biochemically characterized BAHDs to the newly identified BAHDs from each species. Here, we used blastall with the following parameters: *-p blastp -e 1 -m 8*. Subsequently, we filtered out the best top hits and applied a filter of 40% sequence identity and 200 amino acid match length between query and target. In approach 2, we used phmmer (hmmer v3.3 package) (Potter et al., 2018) with the following parameters: *–noali -E 1e-20.* Afterwards, we filtered out hits with e-value larger than 1e-50. For comparison, we also ran phmmer without specified e-value. Finally, we used ITOL v.5.6.2. (Letunic and Bork, 2021) to map the clade assignments to the individual, species-specific BAHD trees to illustrate the spread of each clade across the analyzed species.

### 2.5 Gene expression analysis in Arabidopsis and rice and calculation of synonymous rate

Normalized gene expression information for Arabidopsis was downloaded from Arabidopsis RNA-seq database (ARS; http://ipf.sustech.edu.cn/pub/athrna/) (Zhang et al., 2020). Expression data for rice was gathered from: https://tenor.dna.affrc.go.jp/downloads (downloaded on June 4, 2021). Subsequently we translated RAP-DB locus IDs to MSU locus IDs using a mapping file downloaded from https://rapdb.dna.affrc.go.jp/download/irgsp1.html (downloaded on June 4, 2021). Expression values of BAHDs were isolated from each of the datasets, and were used to calculate Pearson correlation coefficient using base R v4.0.5 (R Core Team, 2021). Plots were generated in R using ggplot2. The blue line represents the best fit of a linear model (lm) and the shaded area represents the 95% confidence interval. R^2^ was calculated using the lm formula in base R (R Core Team, 2021). Kruskal-Wallis rank sum tests were performed to detect significant differences using entire datasets using base R. Statistical tests between bins were performed using Kolmogorov-Smirnov (KS) test with multiple testing correction using p.adjust (method = “fdr”) in R. All tests were performed in R v4.0.5 (R Core Team, 2021). For calculating substitution rates, all pairwise comparisons of paralogous BAHDs were calculated using the yn00 function in the PAML software and compiled using custom Python scripts.

### 2.6 Co-expression analysis for pathway prediction

The ATTED co-expression data table (Ath-u.v21-01.G18957-S27427.combat_pca_subagging.ls.d) was downloaded (Obayashi et al., 2022). This table was constructed using integrative assessment of both RNA-seq and microarray datasets as described here (https://atted.jp/static/help/download.shtml#method). The co-expression significance value of each gene is expressed as a z-score. From the Entrez Gene IDs noted in the ATTED data file, genes with z-score ≥ 3 were considered co-expressed while those with z-score ≤ 1 were considered not-co-expressed. Pathway assignments for each gene were obtained from Plant Metabolic Network (ara_pathways.20210325.txt) (Hawkins et al., 2021). For each BAHD, we first asked which other genes with pathway information were co-expressed. Using the co-expressed and not-co-expressed gene sets, we then performed an enrichment analysis to determine if a given pathway was enriched among the co-expressed genes. Statistical significance was determined using Fisher Exact Test with multiple testing correction based on Q-value (Storey, 2002).

## 3 Results

### 3.1 BAHD gene and protein features experienced substantial changes in land and non-land plant lineages

**Figure 1:**
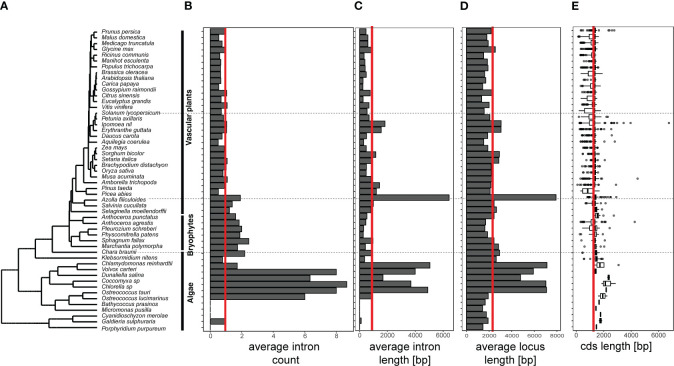
Genomic structure of BAHDs accross 52 representative genomes of plant species. **(A)** Representative species were selected from algal and land plant species and a species tree was inferred and illustrated as described in Kruse et al., 2022 and Moghe et al., 2023. Subsequently, we identified BAHDs using hmmsearch, estimated the number of introns per gene **(B)**, intron length **(C)**, locus length **(D)**, and coding sequence (cds) length **(E)** using custom Python scripts. The vertical red line indicates the average across all analyzed species.

Previous results demonstrated that BAHDs expanded in land plants faster than the increase in genomic gene content *via* repeated duplications (Kruse et al., 2022). Investigation of 52 sequenced genomes revealed that this expansion was also associated with changes in gene and protein structure. The number of BAHDs increased from 1-5 copies in algal genomes to dozens to hundreds of copies in diploid plant genomes (Kruse et al., 2022). An interesting gradation in intron counts was observed (**Figures 1A, B**), with chlorophyte BAHDs having multiple introns, non-seed plant BAHDs generally showing a single intron, and most seed plant BAHDs having 0-1 introns. Furthermore, for chlorophytic BAHDs with introns, the average intron size is also considerably larger than expected (and for unknown reasons, the fern *Azolla filiculoides*), leading to larger overall gene locus length (**Figures 1C, D**). It is not clear if the *A. filiculoides* intron size increase is associated with the overall high number of transposable elements in the genome (Li et al., 2018). On average, the coding sequence length of the algal BAHDs is also larger (**Figure 1E**), indicating that these BAHDs may have different roles and regulatory behaviors than angiosperm BAHDs.

To investigate whether algal BAHDs have different domain structure than land plant BAHDs, we scanned all identified BAHDs for presence of any other domains as described in the Protein Family (PFAM) annotation. The PFAM database identifies 285 different architectures for this domain model (PF02458) across 791 species, with 88.2% of BAHDs not showing co-occurrence with any other domain. The algal genomes also follow this trend. We specifically analyzed 38 BAHD sequences from 28 species, of which 28 (73.6%) sequences showed a singular BAHD domain. This result suggests that most functional novelty in algal BAHDs arises due to innovations within the BAHD domain as against due to its co-occurrence with other domains. Furthermore, alignment of 13 algal BAHDs selected previously (Kruse et al., 2022) with twenty random, biochemically characterized land plant BAHDs revealed that eight BAHD algal sequences (e.g. from *Chlamydomonas reinhardtii*, *Chara braunii*, and *Micromonas pusilla*) were longer and contain sequence regions that cannot be found in land plant BAHDs, the significance of which is not clear (**Supplementary Figure 1**). To the best of our knowledge, only one BAHD from algae (*Chara braunii* HQT-like) has been characterized (Kruse et al., 2022). When tested *in vitro* against a panel of 12 substrates, this enzyme catalyzed only the acylation of quinate using coumaroyl-CoA, however, it is unknown if other *in vivo* substrates of this enzyme exist. CbHQT-like, despite its marked longer sequence length (572 residues) compared to the average length of land plants (407 residues), catalyzes a typical BAHD reaction that is conserved across land plants. Currently the explanation for structural differences of algae and land plant BAHDs remains unknown.

The BAHD family was previously predicted to have expanded in land plants, resulting in seven different clades (Kruse et al., 2022; Moghe et al., 2023). These clades are functionally divergent, with their functions defined using experimentally characterized BAHDs belonging to those clades. Thus, we explored the clade-wise expansions of BAHDs over multiple species over land plant evolution.

### 3.2 Clade-specific expansions and duplication-divergence characterize BAHD evolution in land plants

**Figure 2:**
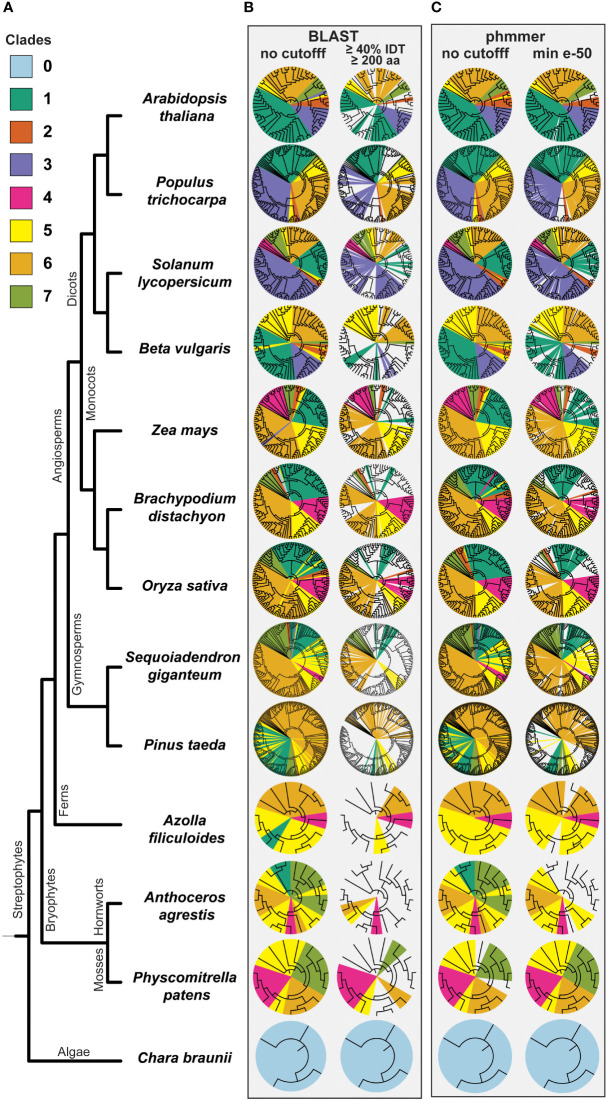
Phylogenetic distribution of BAHD clades in selected plant species. **(A)** Representative species were selected from early diverging plant lineages, gymnosperms, monocots, and dicots. Clade colors correspond to previously published clade assigne-ments of characterized BAHD members (Kruse et al, 2022). BAHD sequences were assigned to clades using **(B)** blastp with either no cutoff or a 40% IDT, 200 aa match length cutoff and **(C)** using the software phmmer with no cutoff or a minimum e-value of e-50. Each phylogenetic tree was inferred using IQTree with 1000 non-parametric bootstrap replicates, illustrated in iTOL (Letunic and Bork, 2021) and mapped clades were indicated using different colors.

To better understand the expansions of the seven clades in land plants, we obtained maximum likelihood trees of BAHD protein sequences from 13 species selected in a phylogeny-guided manner. Using BLAST and phmmer, we identified the best hits of each BAHD in a given species to the biochemically characterized enzymes previously defined to be in each clade (Kruse et al., 2022; Moghe et al., 2023), adopting a clade nomenclature that was updated from a system used earlier (D’Auria, 2006; Tuominen et al., 2011). Two versions of the similarity search results are shown (**Figure 2**) – the top hits of BLAST and phmmer (which is more sensitive than BLAST) without any filtering (Relaxed Set), and hits after filtering them with a 40% identity and 200 amino acid length threshold that, in our experience, typically filters random hits of BAHDs (Conservative Set). While the Relaxed Set assigns every enzyme in each species to a clade, the Conservative Set reveals novel, species-specific clades comprising BAHDs that have sufficiently diverged from their ancestors at the sequence level. Although there were slight differences between BLAST and phmmer, the overall trend remained the same.

The *C. braunii* BAHDs are sufficiently divergent from land plant BAHDs, forming a separate clade on their own, previously referred to as clade 0 (Kruse et al., 2022). Clade 1, involved primarily in anthocyanin and flavonoid acylation, is only detected in the Conservative Set within seed plant genomes. This observation is congruent with emergence of the anthocyanin biosynthetic pathway – in which BAHDs catalyze the last decoration steps – in seed plants, primarily angiosperms (Piatkowski et al., 2020) and agrees with our previous OG-based inference (Kruse et al., 2022; Moghe et al., 2023). Clade 2, involved in wax biosynthesis, is seen in the Conservative Set-in only angiosperm plant clades. Clade 3, involved in acylation of diverse chemical scaffolds such as sugars, flavonoids and alkaloids was restricted to dicots, with no high-confidence hits found in other species groups. The absence of clade 3 in other plant genomes than eudicots suggests that Clade 3 is primarily a dicot-specific innovation. However, additional sampling of plant genomes from monocots, outside of Poaceae, and further early diverging eudicots would be needed to confirm Clade 3 exclusivity to dicot plants. Interestingly, Clade 4, associated with amine acylation, was found to be expanded in monocot grasses compared to the sampled dicot species, possibly reflecting the high prevalence of phenolamides (hydroxycinnamic acid amides) in grasses (Peng et al., 2016; Roumani et al., 2021). Surprisingly, no members of this clade were detected in the gymnosperm *Pinus taeda* (loblolly pine), beets, poplar and Arabidopsis, the significance of which is not clear. Clades 5 and 6, involved in aromatic alcohol acylation (e.g. in phenylpropanoid biosynthesis) and aliphatic alcohol/terpene acylation are present in all land plants, consistent with the role of these building blocks in the conquest of land. As suggested by previous studies, most of the biochemical and genetic pre-requisites for the evolution of true lignin and other derivatives of the general phenylpropanoid pathway were already present in the common ancestor of land plants (Weng and Chapple, 2010; Espiñeira et al., 2011; Renault et al., 2019; Kriegshauser et al., 2021; Rencoret et al., 2021). Our previous results (Kruse et al., 2022) also suggested that the ability to produce caffeoylquinate – the hydroxycinnamoyl CoA quinate transferase (HQT) activity – exists in charophytic algae and likely existed in the ancestor of land plants.

We also surveyed BAHDs in sugar beet, which produces betalain alkaloids. Betalains are produced due to a diversion of flux from arogenate, away from flavonoid and anthocyanin production (Lopez-Nieves et al., 2018; Timoneda et al., 2019). Thus, we expected Clade 1, involved in anthocyanin and flavonoid acylation, to have contracted in that genome. In contrast, in poplar and giant sequoia – both woody tree species – we expected BAHDs involved in lignin production (clade 5) and generally, aromatic alcohol acylation, associated to terpenoid production to have increased in number. No such trends were observed (**Figure 2**).

The Conservative Set also revealed potential novel clades in bryophyte genomes (**Figure 1B**). The sampled bryophytes have 21 and 15 BAHDs, but the *in vitro* activities and physiological roles of most of these novel BAHDs have not been characterized. Such unassigned clades are also seen in other non-seed plant genomes and likely reflect the relative disparity in BAHD research in these clades. While it is possible that enzymes belonging to these clades have the same or similar activities to already known BAHD clades, at the sequence level, they have diverged substantially from any characterized BAHD enzyme to be assigned to a known clade using our clade-assignment approaches and thresholds. Such diverged clades are also seen in every other angiosperm and gymnosperm species including *A. thaliana*, highlighting the continuous functional innovation that occurs *via* duplication in this family.

### 3.3 Multiple motifs exposed to substrate binding cavities are enriched in BAHD clades

**Figure 3:**
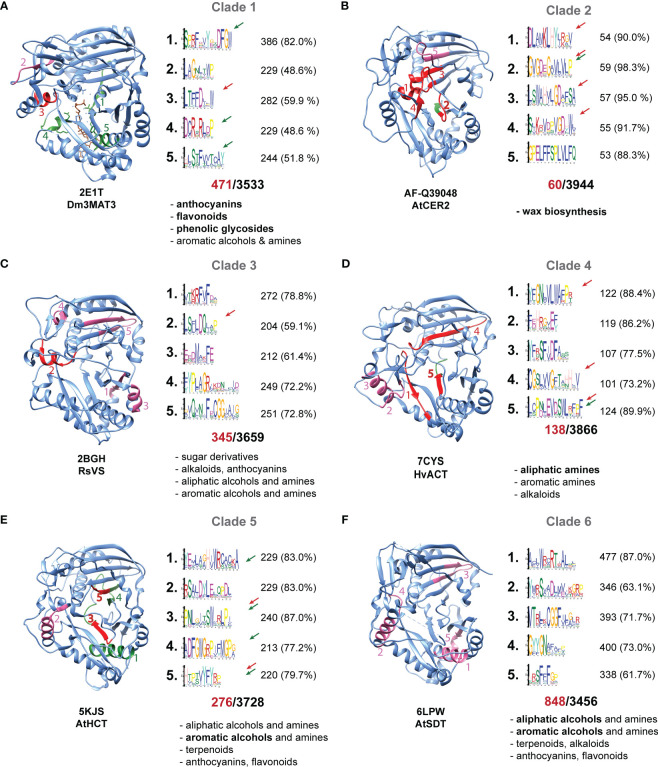
Clade-specific enriched motifs. **(A–F)** Motifs that are enriched in the sequences in the OGs of the respective clade, in comparison to all other OGs identified across 52 representative plant genomes. Motifs were identified using STREME in discriminatory mode. Only the top 5 enriched motifs are shown, and their locations on the protein structures are highlighted. Clade 6 motif locations are best guesses since some motifs are not found in AtSDT. Each protein structure is positioned with the acceptor binding pocket in the foreground and donor CoA binding pocket in the back. Motifs that are exposed to the acceptor side are highlighted with a red arrow and colored red within the structure. Motifs exposed to the donor side indicated are indicated with a green arrow and colored green. Structural motifs are colored in pink. For each clade, the number in red below the motifs indicates the number of sequences found in the corresponding OGS of that clade, and the black number indicates the number of sequences used as background. The number and percentage of sequences with the motif are given for each motif. All known substrate classes of each clade are noted, according to the nomenclature introduced in Kruse et al., 2022, with the most typical structural class bolded.

Identification of unique motifs may help in functional prediction of BAHDs. For example, DFGWG and HXXXD are two distinguishing and functionally important motifs of the family, and different variations of the adjoining residues appear in different clades (**Figure 3**). These motifs are structurally and catalytically important, respectively, with the His residue playing a key role in catalysis. To extend such insights, we asked if specific motifs were enriched among individual BAHD clades, using the sequences of biochemically characterized BAHDs in that clade vs. all other enzymes not in that clade. To ensure enough sample size for this discriminant analysis, we boosted the numbers of the BAHDs wherever required using orthologous groups of those enzymes as previously defined (Kruse et al., 2022). Several clade-specific motifs were discovered.

For each enriched motif, we asked if it was structural or likely-important for substrate binding/catalysis, based on whether the side chain of at least one residue in the motif was exposed to the acceptor/donor binding pocket. Clade 1 enzymes are prominently involved in malonyltransferase reactions whereas all other clades typically catalyze acyltransfer using aromatic (coumaryl, feruloyl, benzoyl CoA) or aliphatic (C2-C12 carbon CoA) donors. We found that 3/5 enriched motifs in this clade were located exposed to the donor CoA binding pocket. The motif TFFDXXW was also found to be enriched. Through site-directed mutagenesis and molecular dynamic simulations, we previously identified the role of the Trp residue in positioning the anthocyanidin core for acylation (Kruse et al., 2022). Another motif YFGNC, which is enriched in subclade 1a/b involved primarily in anthocyanin/flavonoid acylation (**Supplementary Figure 2**), was not found to be differentially enriched when assessing clade 1 as a whole, likely due to substantial functional divergence of clade 1c/d in comparison to the anthocyanin/flavonoid acylating enzymes of clade 1a/b. Aligning sequences belonging to clade 1a/b with clade 1c and 1d shows that the Cys residue, important for anthocyanin/flavonoid activity (Kruse et al., 2022), does not occur in 1c and 1d, suggesting this residue’s close association with the anthocyanin/flavonoid acylating activity (**Supplementary Figure 2**).

In contrast to Clade 1, Clade 2 enzymes involved in wax biosynthesis had 4/5 enriched motifs located exposed to the *acceptor* binding cavity, of which one also extended in the donor binding site. This is a unique clade involved in long-chain fatty acid/alcohol acylation, for which no biochemical *in vitro* activities are available. Due to the unique hydrophobic nature of their acceptor substrates, it is likely that these motif changes reflect the acceptor site remodeling that may have occurred in these enzymes.

Clade 3 is a multifunctional, rapidly diverging clade (D’Auria, 2006; Tuominen et al., 2011; Kruse et al., 2022; Moghe et al., 2023). We see only one motif exposed to the *acceptor* binding site. The rapid divergence of these enzymes – as previously seen by the long branch lengths in a tree of characterized BAHDs (Kruse et al., 2022) – may have led to lack of any commonly enriched motifs in this clade. In contrast, Clade 4 (amine acylation) and Clade 5 (aromatic alcohol acylation), contain three and four motifs respectively that are exposed to the acceptor or donor binding pockets. Activities of Clade 6 are, overall, very similar to Clade 5 and both clades appear across all land plants (**Figure 2**). However, no enriched motif was found exposed to the acceptor/donor binding sites. Most of the enriched motifs in Clade 6 are likely structural in nature.

In Clades 4,5 and 6, different variations of the SXXD motif were differentially enriched. This motif is not in the active site or exposed to the substrate binding pockets but is part of a helix. The role of this motif is not clear, however, given its position and proximity to loops, we postulate that this motif acts as a hinge and may be involved in allosteric movement of the protein upon donor CoA binding, influencing the specificity of the acyltransferase reaction. Indeed, a previous study (Levsh et al., 2016) identified AtHCT Arg356 – which is just 4 aa away from the enriched SXXD motif in AtHCT – as an important driver of substrate selectivity in Clade 5 HCT enzymes. The entire motif, however, may play a key role in this allosteric movement.

This analysis was restricted at the clade level to obtain enough sample size for performing the discriminant analysis. It is possible that sub-clade-specific analyses – in some sub-clades such as clades 1a/b and 5a (**Supplementary Figure 3**), or clades 6c, 6a – may reveal additional function-specific motifs. Nonetheless, the motifs identified in this analysis that are exposed to the acceptor or donor sites are attractive targets for site-directed mutagenesis to enable activity engineering of these enzymes.

While sequence level information can provide insights on the ability of an enzyme to use a given substrate class, the actual substrates being used depends on the cellular localization of the enzymes. While sub-cellular and cell-type specific data is limited, analysis of condition-wise and organ-wise expression data can provide independent insights about BAHD evolution. Therefore, we first explored previously compiled expression data in rice and Arabidopsis to answer questions regarding evolution of paralog BAHD expression profiles.

### 3.4 Most BAHD paralogs have diverged substantially in their expression patterns in Arabidopsis and rice

**Figure 4:**
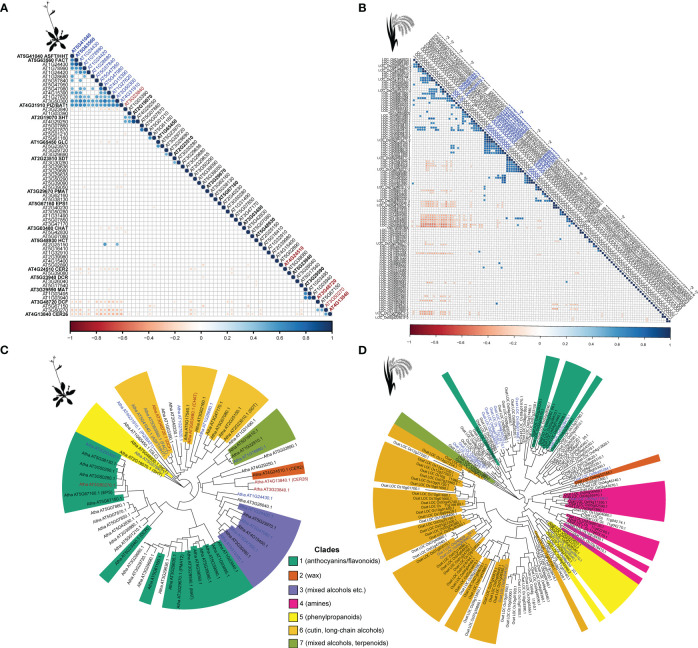
Co-expression of BAHDs in rice and Arabidopsis. **(A)** Co-expression matrix of Arabidopsis BAHDs. **(B)** Co-expression matrix of rice BAHDS. **(C)** Maximum-likelihood tree of Arabidopsis BAHDs. **(D)** Maximum-likelihood tree of rice BAHDS. Trees were inferred using IQTREE with 1000 bootstrap replicates and different colors represent different clades that were mapped using BLASTP (see Methods). Blue colored sequences were found to be positively coexpressed (PCC >= 95th percentile) and sequences colored in red are strongly negatively correlated (PCC <= 5th percentile). Sequences highlighted in bold in **(A)** have functional annotations. Clade nomenclature corresponds to the nomenclature introduced in Kruse et al., 2022 and typical substrate classes (but not all) are noted for each clade.

**Figure 5:**
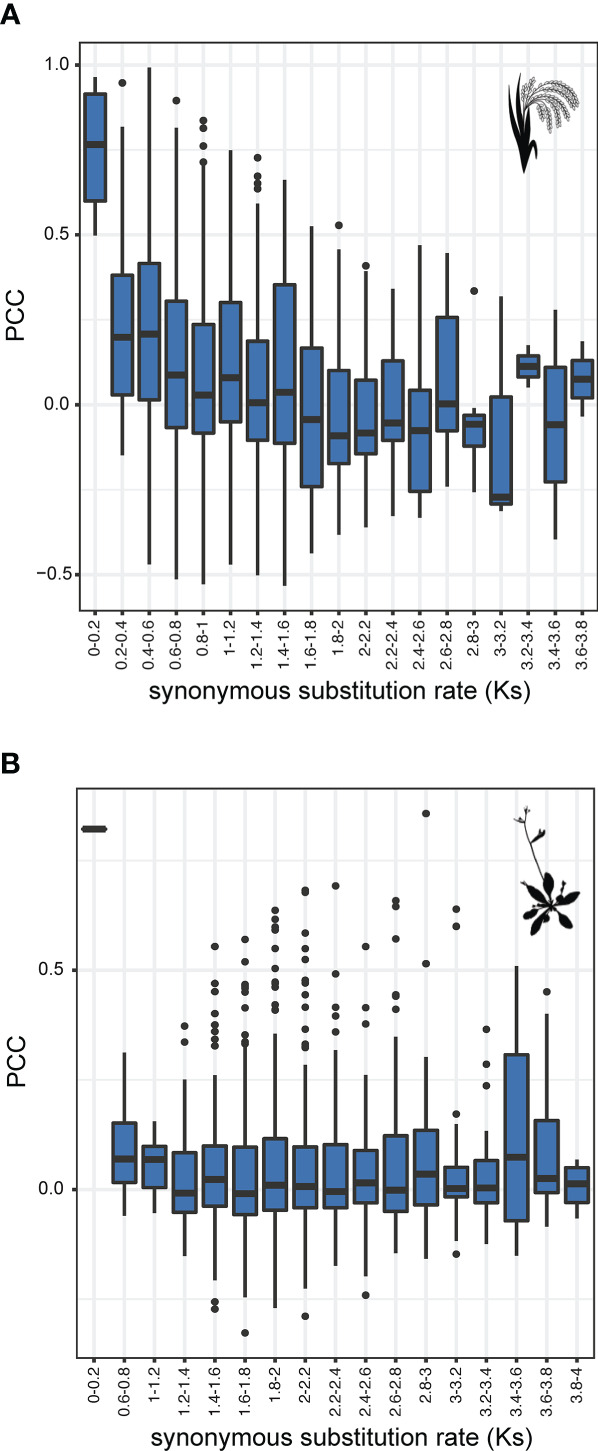
Relationship between synonymous substitutions (Ks) and expression correlation of BAHD paralogs. Expression correlation (PCC) versus synonymous substitution rate (Ks) of **(A)** rice and **(B)** Arabidopsis BAHDs. No significant differences were found when comparing across the entire dataset (Kruskal-Wallis rank sum test p=0.121 and p=0.494, respectively). However, comparing individual Ks bins for rice using pairwise Kolmogorov-Smirnov tests revealed significant differences between bin 0-0.2 in several bins up to bin 2.6-2.8 (significance level 0.05).

Diversification of an enzyme family can occur both at sequence and expression level. To determine how annotated BAHDs are different in their functions, we studied normalized RNA-seq data from previously published studies in *Oryza sativa* (rice) and *Arabidopsis thaliana* (Arabidopsis). In the latter, 18,916 expression data points for each of the 64 BAHDs were used to calculate pairwise Pearson’s Correlation Coefficients (PCC), while in rice, 136 data points were used for 115 BAHDs. In both species, most BAHDs are uncorrelated with each other, however, multiple pairs were found to be significantly positively and negatively correlated with each other, at >95^th^ and <5^th^ percentile respectively of the overall correlation distribution (**Figures 4A, B**), highlighted in blue and red, respectively). We asked if these highly correlated genes tend to be recent gene duplicates. Mapping the largest correlated cluster onto the gene tree did not suggest any specific clustering in Arabidopsis (**Figures 4C, D**), however, five pairs of recently duplicated genes were found to be correlated in rice. These genes belonged to aromatic alcohol acylating (1 pair), amine acylating (1 pair) and rice-specific clades with unknown function (3 pairs). Synonymous substitution rate (Ks) between BAHDs, which is a proxy for their time since duplication, did not explain the variation in PCC (R^2^: 0.04 and -0.0002 in rice and Arabidopsis, respectively) (**Supplementary Figures 4A, D**). Similarly, non-synonymous substitution rate (Ka) and the Ka/Ks also did not explain the variation in PCC (**Supplementary Figures 4B–F**). This was likely due to an overabundance of highly diverged paralogs biasing the regression (**Supplementary Figure 4**). Splitting paralogs into Ks bins revealed that only the most recent BAHD paralogs (0<Ks<0.2) in rice were significantly more co-expressed than BAHDs in other bins (Kolmogorov-Smirnov test, corrected p-value < 0.06, **Supplementary File 1**). This trend was not observed in Arabidopsis due to lack of paralogs in this Ks bin. In both species, a trend quickly became undetectable beyond Ks>0.4, suggesting that the regulation of BAHD expression changes rapidly after duplication (**Figures 5A, B**), corroborating earlier studies on expression divergence of gene duplicates (Ganko et al., 2007; Renny-Byfield et al., 2014). These results suggest that co-expression between BAHDs may rarely be informative of function.

### 3.5 Predicting BAHD functions using co-expression information

Above correlation analysis showed that most BAHDs have diverged significantly in their expression patterns from their paralogs. In our previous study (Kruse et al., 2022), we used orthology as a means to predict *in vitro* substrate utilization patterns of BAHDs and demonstrated that this approach successfully predicted correct substrate classes in 80-92% instances where a class could be assigned. To determine if co-expression with other genes can be used to obtain orthogonal evidence for *in vivo* function and help prioritize candidates for downstream experimental analyses, we investigated co-expression patterns of Arabidopsis BAHDs.

We first identified all co-expressed genes for each BAHD using ATTED – a pre-compiled database containing co-expression data for 18,957 Arabidopsis genes (Obayashi et al., 2022). In parallel, Plant Metabolic Network (PMN) pathway database was used to map all Arabidopsis genes to specific metabolic pathways. Overall, 2714 Arabidopsis genes in the ATTED database were assigned to PMN pathways. For each BAHD, we first identified all co-expressed and not-co-expressed genes mapped to PMN pathways (see Methods), and then asked if any of the matching PMN pathways were enriched among the co-expressed genes (Fisher exact test, corrected p-value < 0.05). Of the 65 BAHDs in Arabidopsis, 33 (50.7%) were previously assigned to at least one pathway in PMN (**Supplementary File 2**) and enrichment test could be performed for 33/65 BAHDs that were present in both ATTED and PMN databases and had highly co-expressed genes. Of these, 27 BAHDs had at least one enriched PMN metabolic pathway. We note that co-expression results may not necessarily identify the pathways a given BAHD is involved in, for example, because the actual pathway is not known, not enough/too many enzymes are mapped to the given pathway to reveal significant co-expression signal or because the biological phenomenon where the BAHD is expressed in may result in many co-expressed pathways. Furthermore, spurious correlations due to above and/or other technical reasons may lead to misassigning of genes to metabolic pathways in which they are not involved. Therefore, the co-expression results were primarily used to identify the overall metabolic class or the biological process a given BAHD may be associated with, instead of the actual *in vivo* pathway or specific substrates/molecular function. These co-expression based predictions were then combined with orthology-based predictions obtained previously (Kruse et al., 2022) to obtain greater confidence in the functional predictions.

We first asked if the co-expressed processes of the known BAHDs matched expectation, followed by assessment of other BAHDs showing similar prediction patterns. Known enzymes involved in lipid and cuticular wax/suberin biosynthesis such as ECERIFERUM 2 (CER2), DEFICIENT IN CUTIN FERULATE (DCF), PERMEABLE LEAVES 3 (PEL3), REDUCED LEVELS OF WALL-BOUND PHENOLICS 1 (RWP1), FATTY ALCOHOL : CAFFEOYL COENZYME A ACYLTRANSFERASE (FACT) were correctly predicted as being involved in lipid metabolism pathways such as those of suberin, cutin, very long chain fatty acid and acylglycerol biosynthesis/degradation (**Supplementary File 2**). DCF and PEL3 were also assigned “aliphatic alcohols” as the *in vitro* substrate class. AT3G23840 and AT5G02890 (both unannotated) were also significantly co-expressed with genes mapped to lipid metabolism, and therefore may be involved in similar processes. The former was mapped to the orthologous group of AtCER2, providing further support to its prediction, while only co-expression-based result is available for the latter candidate. Another characterized enzyme ACETYL COA: (*Z*)-3-HEXEN-1-OL ACETYLTRANSFERASE (AtCHAT) (D’Auria et al., 2007a) – known to be involved in 3-hexen-1-yl acetate biosynthesis – was co-expressed with other enzymes in the same pathway as well as those in cytokinin and chlorophyll degradation, the significance of which is not known. AT5G17540 received similar *in vitro* substrate class annotations but did not have any co-expressed pathways. This enzyme was previously shown to influence brassinosteroid metabolism (Zhu et al., 2013).

AT3G29590 [MALONYL COA: ANTHOCYANIDIN 5-O-GLUCOSIDE-6’’-O-MALONYLTRANSFERASE; At5MAT, (D’Auria et al., 2007b)] is involved in anthocyanin acylation and was correctly mapped to the same and related pathways using co-expression. Additionally, AT1G03940/3495 (both unannotated) were also significantly co-expressed with anthocyanin-biosynthetic enzymes. Based on our substrate class prediction algorithm (Kruse et al., 2022), these enzymes are predicted to use “anthocyanins/flavonoids/phenolic glycosides”, which agrees with their co-expression patterns. *A. thaliana* PHENOLIC GLUCOSIDE MALONYLTRANSFERASE (AtPMAT1) is involved in acylation of phenolic glycosides and is considered a detoxification enzyme (Taguchi et al., 2010; Gan et al., 2021). Consistent with its role, it was predicted to use “flavonoid” class (which as per previous definition (Kruse et al., 2022) also includes phenolic glycosides) and was found to be co-expressed with not only other phenolic glucoside pathway enzymes but also with several enzymes involved in flavonoid biosynthesis, detoxification of reactive carbonyls, glutathione-mediated detoxification, flavonoid biosynthesis and abscisic acid pathways (**Supplementary File 2**). Based on its phylogenetic position, its substrate class is predicted to be anthocyanins/flavonoids/phenolic glycosides, which also agrees with its role. No other BAHD, however, showed similar patterns.

Characterized genes such as AtPMAT2 (also involved in phenolic glycoside biosynthesis), AtBIA1/DRL1 (involved in brassinosteroid biosynthesis (Roh et al., 2012; Zhu et al., 2013), AT2G240230 (DRL1 homolog) could not be confirmed because they either did not have a significantly co-expressed PMN pathway, *in vitro* class prediction or both. The predictions for *A. thaliana* SPERMIDINE HYDROXYCINNAMOYLTRANSFERASE (AtSHT) (Grienenberger et al., 2009; Wang et al., 2021), were incorrect using both methods.

*A. thaliana* ENHANCED PSEUDOMONAS SUSCEPTIBILITY (AtEPS1) (Torrens-Spence et al., 2019) involved in salicylic acid metabolism, is highly co-expressed with glucosinolate-biosynthetic enzymes. This may be explained by the involvement of both compound classes in defense responses e.g. by salicylic acid inducing glucosinolate accumulation (reviewed in Halkier and Gershenzon, 2006; Textor and Gershenzon, 2009). Multiple BAHDs (11/33, 33%) were annotated in PMN to be involved in simple coumarins and chlorogenic acid biosynthesis. The co-expression analysis predicted defense response roles to many of these BAHDs (AT3G50280, AT5G67150, AT3G50270) due to their co-expression with flavonoid, glutathione-mediated detoxification, glucosinolate and jasmonate biosynthetic pathways (**Supplementary File 2**).

These results suggest – based on functional analysis of previously characterized enzymes – that combining co-expression with pathway models and *in vitro* activity based predictions can generate useful preliminary hypotheses about BAHD roles in specific metabolic pathways and/or biological processes. While not all co-expression-based predictions are accurate, combining them with orthology-based predictions can help increase confidence in the BAHD’s biochemical function. Nonetheless, further wet-lab characterization is required to validate functional predictions of the yet-uncharacterized enzymes.

## 4 Discussion

Processes inherent in the evolution of enzyme families – gene duplication-divergence, promiscuity, allelic divergence – are some of the biggest drivers of metabolic diversification in plants (Weng et al., 2012; Weng, 2014; Copley, 2015; Moghe and Last, 2015; Copley, 2020; Copley, 2021). A better understanding of these processes can help improve models for functional prediction of enzymes involved in metabolism (de Crécy-lagard et al., 2022). In this study, we sought to determine how the large BAHD acyltransferase family has evolved in plants (**Supplementary File 3**), and whether there are any sequence and/or expression features that can aid functional prediction.

We found that only ~1-5 BAHDs may have existed in the common ancestor of land plants and algae but their numbers quickly increased in land plants (Kruse et al., 2022) with a concomitant change in gene structure, producing shorter coding sequences and typically fewer introns than algal sequences. It is not clear, however, what the ancestral state for the gene structure was. Both charophytic algae (*C. braunii, K. nitens*) show shorter introns and genetic loci than chlorophytic algae. Therefore, it is also possible that the intron size, locus length, CDS length and number of introns increased in chlorophytic algae. The significance of these differences is unknown, especially since no BAHD functions and structure-function studies have been reported from chlorophytic algae.

In land plants, our results show that BAHD expansions occurred differently in different species. For example, BAHDs involved in phenylpropanoid biosynthesis and aliphatic alcohol acylation are present across all land plants (and therefore likely ancestral). However, BAHDs orthologous to known amine acylating enzymes such as agmatine coumaroyltransferase and spermidine coumaroyltransferase – despite their orthologous group being present in all land plants –have expanded specifically in monocots (Poaceae) (**Figure 2**). Hydroxycinnamic acid amides (HCAAs) are known to be important in grasses for pest and pathogen defense as well as for maintaining cell wall integrity (Roumani et al., 2021). Homologs of the characterized N-acyltransferases are also over-represented among BAHDs in *Physcomitrella patens* (moss) – it would be interesting to assess what roles these enzymes play in mosses and whether they too have the N-acyltransferase activities. Similarly, Clade 3 – which is involved in multiple specialized metabolic pathways – is likely dicot-specific and has been crucial in evolution of new metabolic classes such as acylsugars, alkaloids such as capsaicin, vinorine and cocaine, triterpenoids e.g. thalianol and arabidiol, and several anthocyanin acylating activities. In addition, we identified several clades whose members have sufficiently diverged from experimentally characterized enzymes to be placed into known clades. This is especially true in ferns and bryophytes but also true in other species. Such unassigned clades, which could either be an artefact of our technical thresholds or could indeed represent novel BAHD activities, need to be prioritized for functional assays for further understanding of the roles BAHDs play in plant metabolism.

Differential motif enrichment analysis identified unique motifs in the active site, acceptor and donor binding regions, internal structural regions as well as external handles that likely alter protein structure upon donor binding. It needs to be noted that these are simply the enriched regions; the BAHD sequences experience a lot more sequence changes. Despite such perturbations, the overall activity – acylation – has remained essentially the same, pointing to the mutational robustness of the BAHD fold. Residues identified in this study serve as a starting point for more detailed structure-function studies and enzyme engineering in the BAHD family (Ben-Hur and Brutlag, 2006; Kruse et al., 2022).

In addition to sequence features, we asked if expression-based features could be used as predictive signals of function. Due to extensive duplications in the BAHD family, we first sought to understand how expression patterns change with time. Our results suggest that very young duplicates, as expected, have similar expression e.g. while similar expression in more recent rice paralogs (Ks<0.2) is common, there is no differentiation power left at Ks>0.2, indicating background levels of expression divergence are quickly reached in BAHD paralogs. Most characterized BAHDs show substantial substrate promiscuity when assayed *in vitro* (D’Auria, 2006; Kruse et al., 2022; Moghe et al., 2023), suggesting that other mechanisms e.g., altering spatio-temporal expression may play a role in modulating the *in vivo* function of a duplicated BAHD by exposing the enzyme to a different metabolic microenvironment.

While expression similarity between BAHDs has little explanatory power in predicting functional similarity, co-expression with genes in other pathways is indicative of function but needs orthogonal evidences for greater confidence in the prediction. We used Arabidopsis as a test given the wealth of previously characterized enzymes that could be used to test the functional predictions. We found several examples in lipid, anthocyanin, phenylpropanoid and amide biosynthesis where co-expression yielded accurate pathways based on prior knowledge. Most of these predictions were correct, albeit at different levels of functional resolution. The co-expression analysis also yielded novel functional predictions that can be tested using experimental approaches. Such co-expression analysis coupled with orthology-based information to predict *in vitro* and *in vivo* functions, can be significantly more impactful in species where, unlike *Arabidopsis thaliana*, substantial molecular analysis is not possible or has not been carried out before.

Overall, our study provides new insights into the evolution of BAHD acyltransferases, and provides a template to improve BAHD functional annotations. With large enzyme families, selecting impactful targets to characterize and specific hypotheses to test is important. These specifications can help extend our knowledge to clades/parts of the family that are not significantly researched into, and therefore would enable the discovery of novel activities. The functional prediction pipeline outlined in this study – combining expression patterns, pathway knowledge and *in vitro* substrate class prediction – can help to significantly reduce the time to characterize unknown enzymes. Similar approaches can also be applied to other large enzyme families such as CYP450, methyltransferases or UDP glycosyltransferases, expanding our understanding of large enzyme family evolution and providing an impetus to their application in synthetic biology.

## Data availability statement

The datasets presented in this study can be found in online repositories. The names of the repository/repositories and accession number(s) can be found in the article/**Supplementary Material**.

## Author contributions

LK and GM conceived the initial project idea. LK and GM conducted the final analysis and prepared the figures. BF and JC performed initial analysis and developed analysis pipelines. LK and GM wrote the manuscript. BF and JC provided comments, edited and approved the manuscript. All authors contributed to the article and approved the submitted version.

## Supplementary material

Supplementary File 1 Excel file containing the results of the PCC vs Ks analyses.

Supplementary File 2 Excel file containing the results of the co-expression prediction pipeline.

Supplementary File 3 Excel file providing links to the used genome sequence repositories.
