# Supplementary material for: Phylogenomic analyses across land plants reveals motifs and coexpression patterns useful for functional prediction in the BAHD acyltransferase family
Source: Front Plant Sci. 2023 Feb 10;14:1067613. doi: 10.3389/fpls.2023.1067613 (PMC9950517; doi:10.3389/fpls.2023.1067613)
Supplement: Supplementary file 1 [file Image_1.pdf]

Supplementary Figure 1

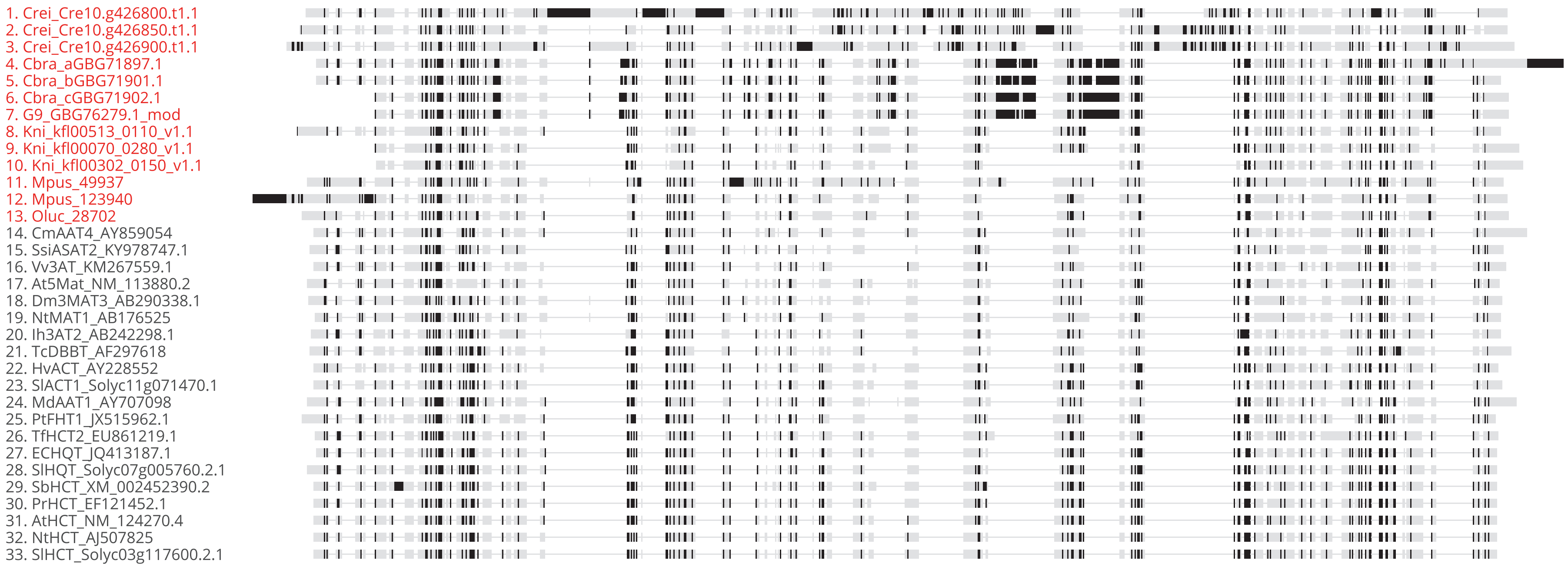

Supplementary Figure 1. Alignment of selected algal BAHD sequences and 20 randomly selected plant BAHDs. The alignment was performed using MUSCLE v3.8.425 and default paramerters in Geneious v2022.1.1. Highlighted in black are agreements to the consensus sequence in at least 50% of the sequences. The percentage is relative to the number of sequences having a residue at a given alignment position.
