# Supplementary material for: Phylogenomic analyses across land plants reveals motifs and coexpression patterns useful for functional prediction in the BAHD acyltransferase family
Source: Front Plant Sci. 2023 Feb 10;14:1067613. doi: 10.3389/fpls.2023.1067613 (PMC9950517; doi:10.3389/fpls.2023.1067613)
Supplement: Supplementary file 2 [file Image_2.pdf]

Supplementary Figure 2

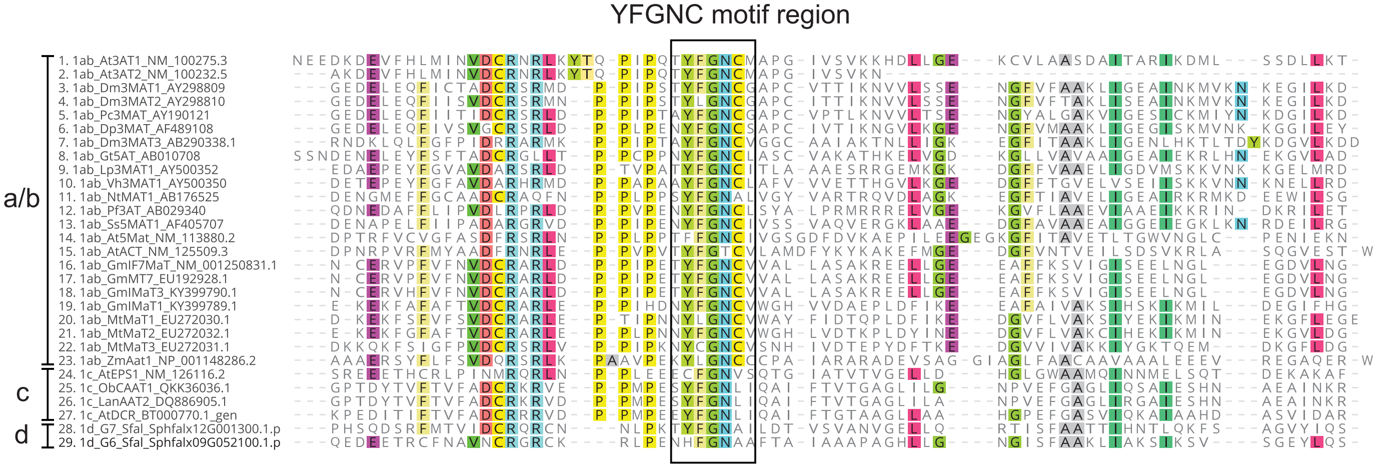

Supplementary Figure 2. Alignment of the YFGNC motif region in clade 1. Biochemically characterized sequences from clade 1 were aligned using MUSCLEv3.8.425 and using default paramerters in Geneious v2022.1.1. The region of the YFGNC motif is highlighted using a black box. Colored residues are conserved in at least 50% of the sequences for each given residue position in the alignment.
