# Supplementary figures and images for: Phylogenomic analyses across land plants reveals motifs and coexpression patterns useful for functional prediction in the BAHD acyltransferase family
Source: Front Plant Sci. 2023 Feb 10;14:1067613. doi: 10.3389/fpls.2023.1067613 (PMC9950517; doi:10.3389/fpls.2023.1067613)

Supplementary Figure 3

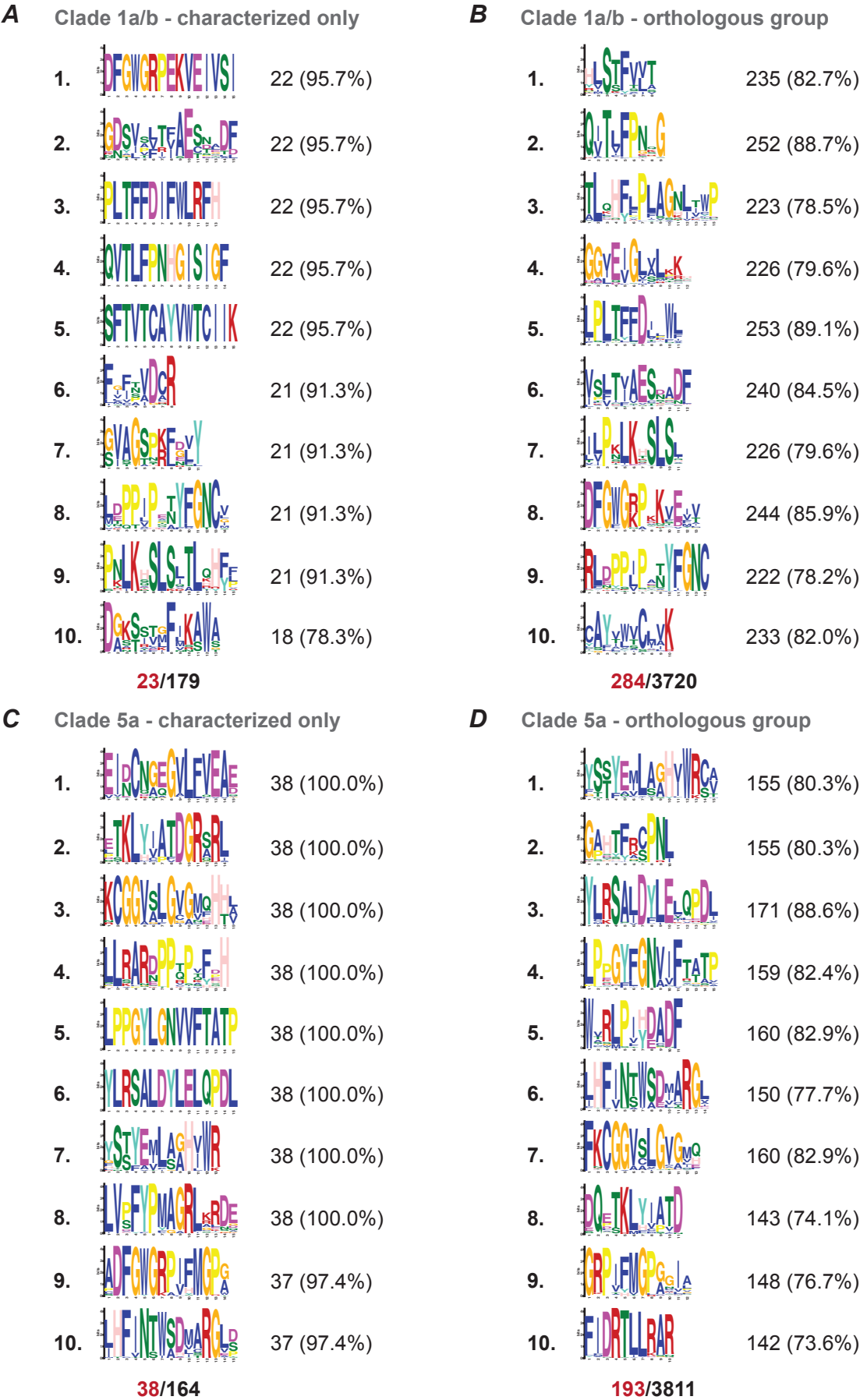

Supplement: Supplementary file 3 [file Image_3.pdf]
