# Supplementary material for: Phylogenomic analyses across land plants reveals motifs and coexpression patterns useful for functional prediction in the BAHD acyltransferase family
Source: Front Plant Sci. 2023 Feb 10;14:1067613. doi: 10.3389/fpls.2023.1067613 (PMC9950517; doi:10.3389/fpls.2023.1067613)
Supplement: Supplementary file 4 [file Image_4.pdf]

Supplementary Figure 4

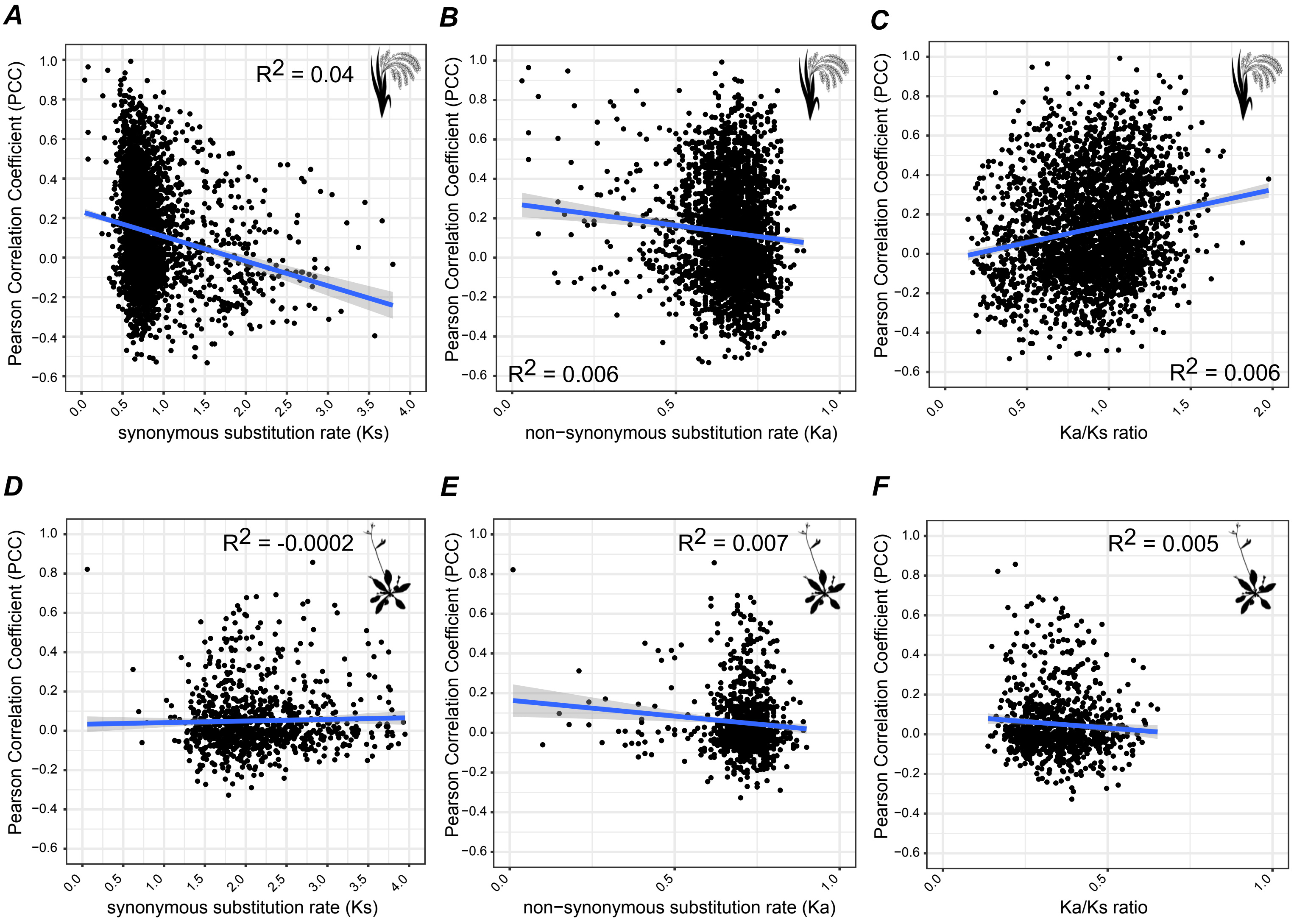

Supplementary Figure 4. Relationship between synonymous substitutions (Ks), non-synonymous substitutions (Ka), Ka/Ks ratio and coexpression of BAHD paralogs. Coexpression of BAHDs (pearson correlation coefficient) versus Ks **(A)**, Ka **(B)**, and Ka/Ks ratio **(C)** in Arabidopsis. Coexpression of BAHDs (pearson correlation coefficient) versus Ks **(D)**, Ka **(E)**, and Ka/Ks ratio **(F)** in rice. Blue line representing the best fit using a linear model and the resulting R^2 are shown. The shaded area of each line represents the 95% confidence interval.
